# Identifying Potential Neoantigens for Cervical Cancer Immunotherapy Using Comprehensive Genomic Variation Profiling of Cervical Intraepithelial Neoplasia and Cervical Cancer

**DOI:** 10.3389/fonc.2021.672386

**Published:** 2021-06-17

**Authors:** Chaohui Bao, Na An, Hong Xie, Ling Xu, Boping Zhou, Jun Luo, Wanqiu Huang, Jian Huang

**Affiliations:** ^1^ Key Laboratory of Systems Biomedicine (Ministry of Education), Shanghai Center for Systems Biomedicine, Shanghai Jiao Tong University, Shanghai, China; ^2^ Shenzhen People’s Hospital, Second Clinical Medical College of Jinan University, Shenzhen, China; ^3^ Department of Obstetrics and Gynecology, Minhang Hospital, Fudan University, Shanghai, China; ^4^ Department of Clinical Laboratory, Jiangsu Health Vocational College, Nanjing, China

**Keywords:** cervical cancer, cervical intraepithelial neoplasia, genome variation, neoantigens, immunotherapy

## Abstract

Cervical cancer (CC) is one of the most common gynecological malignant tumors. The 5-year survival rate remains poor for the advanced and metastatic cervical cancer for the lack of effective treatments. Immunotherapy plays an important role in clinical tumor therapy. Neoantigens derived from tumor-specific somatic mutations are prospective targets for immunotherapy. Hence, the identification of new targets is of great significance for the treatment of advanced and metastatic cervical cancer. In this study, we performed whole-exome sequencing in 70 samples, including 25 cervical intraepithelial neoplasia (CINs) with corresponding blood samples and 10 CCs along with paired adjacent tissues to identify genomic variations and to find the potential neoantigens for CC immunotherapy. Using systematic bioinformatics pipeline, we found that C>T transitions were in both CINs and CCs. In contrast, the number of somatic mutations in CCs was significantly higher than those in CINs (t-test, *P* = 6.60E-04). Meanwhile, mutational signatures analysis revealed that signature 6 was detected in CIN2, CIN3, and CC, but not in CIN1, while signature 2 was only observed in CCs. Furthermore, *PIK3CA*, *ARHGAP5* and *ADGRB1* were identified as potential driver genes in this report, of which *ADGRB1* was firstly reported in CC. Based on the genomic variation profiling of CINs and CCs, we identified 2586 potential neoantigens in these patients, of which 45 neoantigens were found in three neoantigen-related databases (TSNAdb, IEDB, and CTDatabase). Our current findings lay a solid foundation for the study of the pathogenesis of CC and the development of neoantigen-targeted immunotherapeutic measures.

## Introduction

Cervical cancer (CC) is one of the most common gynecological malignant tumors, second only to breast cancer, which occupies the second place in the incidence of women’s malignant tumors in China. There are about half a million new cases of CC every year, and more than 80% of these cases are in developing countries (1, 2). The prognosis of patients with CC is closely related to clinical stage, pathological type, treatment method, patient compliance, etc. Early detection, early diagnosis, and early treatment are the keys to the treatment of CC. Some patients with early cervical cancer can be cured through surgery, chemotherapy, etc. Platinum-based chemotherapy regimens, including cisplatin, carboplatin, fluorouracil, and paclitaxel, are often used in patients with CC (3). However, many patients with advanced cervical cancer and those with metastases to other tissues and organs have a poor prognosis. Hence, the identification of new targets for treatments of CC, especially for immunotherapy, is of great significance for the treatment of advanced and metastatic cervical cancer.

With the development of immunotherapeutic technologies, Immune Check-Point (ICP) inhibitors have been used against different human malignancies, which include metastatic melanoma, bladder cancer, stomach cancer, renal cell cancer, head, neck cancer, etc (4). Compared with the traditional cancer cell inhibition therapies, ICP inhibitors can extend the survival in advanced and metastatic cancer patients who are previously thought to be incurable (5). Up till the present moment, bevacizumab and pembrolizumab are two FDA-approved immunotherapy options for CC treatment, which are only responded in a minority of CC. Therefore, it is vitally important to discover attractive immunotherapeutic targets for CC therapy.

Derived from the somatic mutations, neoantigens only exist on cancer cells, which can have vital roles in tumor-specific T cell-mediated antitumor immunity (6–9). Neoantigens on the cell surface can bind to major histocompatibility complex (MHC) molecules, which can be recognized by T cells and then elicit immune responses (10). It was reported that the patients with high tumor mutation load were apt to benefit from immunotherapies, including PD-L1 and CTLA-4, and other immunotherapeutic technologies (11, 12). Furthermore, it is crucial that neoantigens in CCs can be identified to develop the neoantigen-targeted immunotherapies.

Recently, some studies have analyzed the genomic variations of CC in detail and proved that several mutated genes, such as *PIK3CA*, *PTEN*, *EP300*, *TP53*, *FBXW7*, *MAPK*, *HLA-A*, and *CASP8* (13–17), which are closely related to the occurrence and development of CC. However, the use of genetic mutation information to identify neoantigens of CINs and CCs is limited. Qin et al. used two available public data sets, including The Cancer Genome Atlas cohorts of Cervical squamous cell carcinoma and endocervical adenocarcinoma (TCGA-CESC) data set and the exome and RNA sequencing data [published by Ojesina et al. (13)], to analyze the neoantigen landscape in CC (18). Li et al. investigated cancer epitope trees in early cervical cancer patients by whole-exome sequencing. Each patient displays a unique phylogenetic tree in which almost all subclones harbored neoantigens. These literatures only studied the relationship between mutations and neoantigens in CC (19). However, it is still not quite clear about the landscape of neoantigens during the stepwise process from CINs to CCs.

Herein, we systematically evaluated the genomic variations to identify the potential neoantigens and find better immunotherapeutic targets for CC treatment. We performed a whole-exome sequencing analysis (WES) of CINs and CCs. Our results showed that there were different distributions of the somatic mutated genes with different stages. Our findings could refine our knowledge concerning the occurrence and development of cervical cancer, and were intended to provide attractive immunotherapeutic targets to research and develop the new treatments for cervical cancer.

## Materials and Methods

### Clinical Samples

For physical examination volunteers and patients suffering from CINs and CCs, the written informed consent was obtained from each person before enrollment. The cervical exfoliated cells were sampled using cervical brushes for each enrolled patient, which were used to observe the morphological characteristics based on the liquid-based cytology (Becton Dickinson Company, New Jersey, USA). In Shenzhen People’s Hospital, cervical liquid-based cytological test was used as a routine screening test for CC. Hence, those patients with cytological abnormality were recommended to accept colposcopy examination. And then the colposcopy findings were used to determine if a biopsy is necessary. The diagnoses of CIN1, CIN2, CIN3, or CCs were made and reviewed by two pathologists independently (Smith et al., 2018). This study was approved by the ethical review board of Shenzhen People’s Hospital (SPH-2016010).

A total of 70 samples from 35 patients suffering from CINs and CCs were enrolled in this study. The blood samples from CINs and the paired adjacent paracancer tissues from CCs were used as the control. The biopsies from CINs and the surgically resected tumors and paired adjacent paracancer tissues were snap-frozen in liquid nitrogen and stored at −80°C. Blood samples were stored at −20°C. All tissues were diagnosed by two independent pathologists using hematoxylin and eosin (H&E) staining. The tumor cell content of the analyzed CCs was all more than 75% (ranging from 75% to 90%), while no tumor cells were observed in adjacent tumor tissues and CIN samples (**Supplementary Figure 1**). A detailed description of clinical information was found in **Supplementary Table 1**, which includes source, age, FIGO stage, grade, and pathological type. Besides, there were no significant differences in age among the four groups (Kruskal-Wallis chi-squared test, *P* = 0.06).

### DNA Extraction and Whole-Exome Sequencing

Genomic DNA was extracted from the blood and tissue sample using QIAGEN Blood DNA Mini Kit (Qiagen, Hilden, Germany) and QIAGEN DNA Mini Kit (Qiagen, Hilden, Germany) according to the manufacturer’s protocol. Whole-exome capture was done with Roche SeqCap EZ exome V3.0 (Roche Sequencing, Pleasanton, CA), targeting 64 Mb sequences from exons. Captured libraries were analyzed on Agilent’s 2100 Bioanalyzer. DNA concentrations were measured using Qubit 2.9 Fluorometer (Thermo Fisher, Shanghai, CN). Seventy paired samples on the flowcells were sequenced with Illumina HiSeq X-Ten platform to generate 150-bp paired-end reads. The raw data were trimmed and assembled using Trimmomatic version 0.36 (20) to obtain clean data. Subsequently, the clean data were mapped to the reference human genome GRCh38 using Burrows-Wheeler Aligner (BWA) version 0.7.15 (21) and alignment information was stored in *.sam format. The samtools version 1.3.1 software was employed to converted SAM files into BAM files (http://samtools.sourceforge.net/SAM1.pdf) for each sample (22). Duplicate reads were removed using Picard Tools (http://broadinstitute.github.io/picard/) version 2.17.11 according to the standard data pipeline of the Broad Institute. Local realignments and base quality recalibrations were performed using the Genome Analysis Toolkit (GATK) version 3.8.0 (23).

### Variation Calling Analysis

Somatic SNVs were identified using Strelka (24) algorithm and somatic small insertions or deletions (indels) were detected using VarScan2 (25) algorithm, respectively. All the software was performed for identifying mutations in matched tumor-normal samples with the default parameters. Then, this pipeline was applied as a filter criterion (based on a stringent criterion) to obtain a high-confidence somatic SNVs and Indels. In brief, a mutation was identified as a candidate somatic mutation only when (I) the depth of coverage was no less than 14 in the tumor samples and no less than 8 in the matched control samples; (II) the reads according to the altered allele in the tumor were no less than 5; (III) the fraction of reads according to the altered allele in the tissue was no less than 5% and in the matched control was less than 3%; (IV) the fraction of the reads according to the altered allele frequency in the tumor samples was four-fold or more than in the matched control samples. Finally, mutations were filtered to exclude intronic and silent changes and to retain mutations resulting in missense mutations, nonsense mutations, frameshifts, or splice site alterations. Candidate somatic mutations were further annotated using ANNOVAR (26) for information related to the location, function, previous reports and sequencing data supporting the status of the mutation. Then it converted to MAF files with vcf2maf (https://github.com/mskcc/vcf2maf). The gene functions on mutated genes by using the public database GeneCards (https://www.genecards.org/) (27).

Copy number analysis was performed using CNVkit (28). To obtain copy number alterations in CINs and CCs, genomic identification of significant targets in cancer (GISTIC) 2.0 version 6.2 with join segment size = 4; confidence level = 0.95, and q-value FDR < 0.25 was used (29). Heatmap plots were generated with the MATLAB “colormap” function in GISTIC with segmented Copy Number files.

Somatic mutation signatures were estimated by the Bioconductor package deconstructSigs (30), which identified a linear combination of pre-defined signatures that most accurately reconstructed the mutational profile of a tumor sample. These candidate signatures were compared with the COSMIC signatures. Each mutational signature was assigned a calculated weight representing its contribution to the case samples, where a higher weight value indicates a greater relative contribution of the signature.

Because of the limited numbers of each group sample available for this study cohort, we took a strict enumeration approach to identify potential driver genes. In brief, a gene was considered to be a significantly mutated gene if the following criteria were satisfied: (I) the candidate driver genes were observed in at least 3 out of 35 cases; (II) the candidate driver genes need to be observed in the catalogs of 568 mutational driver genes (31); (III) the candidate significantly mutations were deemed to be deleterious (either a stop-gain or predicted deleterious in two of the three computational approaches applied by SIFT (http://sift.jcvi.org/), Polythen2 (http://genetics.bwh.harvard.edu/pph2/) and MutationTaster (http://www.mutationtaster.org/); The remain mutated genes were considered as potential driver genes.

### HLA Types and Neoantigens Analysis

HLA types for all the patients were performed computationally using POLYSOLVER (32), which used a BAM file as input and employed a Bayesian classifier to determine genotype in four-digit resolution. Likewise, each non-synonymous SNV was translated into a 22mer peptide sequence that centered on the mutated amino acid. Subsequently, the 22mer was separated into 8-11mer *via* a sliding window for detecting MHC class I binding, which was known to be the possible lengths for peptides presented by human MHC class I molecules. We then predicted MHC binding affinity for each peptide as described previously (33). NetMHCpan 4.0 (34) tool was used to determine the binding affinity strength of every mutated peptide to patient-specific HLA alleles for identifying exome-derived neoantigens. And then, the putative neoantigens binders were identified as those with a predicted binding affinity IC_50_< 500 nM and %Rank < 2%. When RNA-seq available, a neoantigen was considered to be expressed if a mutated gene Tumor_FPKM_ ≥ 0.5 and |log_2_(Tumor_FPKM_/Normal_FPKM_)|≥0.1, FPKM, Fragments Per Kilobase per Million.

### Variation Analysis of Public Data

Publicly available somatic mutation profiles were obtained from The Cancer Genome Atlas (TCGA) repositories (https://tcga-data.nci.nih.gov/tcga/), including cervical squamous cell carcinoma and endocervical adenocarcinoma (CESC), head and neck squamous cell carcinoma (HNSC), esophageal carcinoma (ESCA), lymphoid neoplasm diffuse large B-cell lymphoma (DLBC), liver hepatocellular carcinoma (LIHC), ovarian serous cystadenocarcinoma (OV), uterine carcinosarcoma (UCEC), and uveal melanoma (UCS), which were used to analyze the mutation spectrums and mutational signatures related to our results. Raw RNA-seq data that were downloaded from NCBI SRA (SRA315538) were performed to validate the expression of the predicted neoantigens (16).

### Statistical Analysis

All analyses were performed in the R statistical environment version ≥3.5.1. All data were expressed as the mean ± standard deviation (SD) unless otherwise stated. A two-sided Student’s t-test was used to compare quantitative data between two groups and Kruskal-Wallis chi-squared were used to compare categorical data among more groups. Pearson’s correlation coefficient was used to evaluate the linear correlation. *P*-values of <0.05 were considered to be statistically significant.

## Results

### Genomic Alterations in CINs and CCs

**Figure 1:**
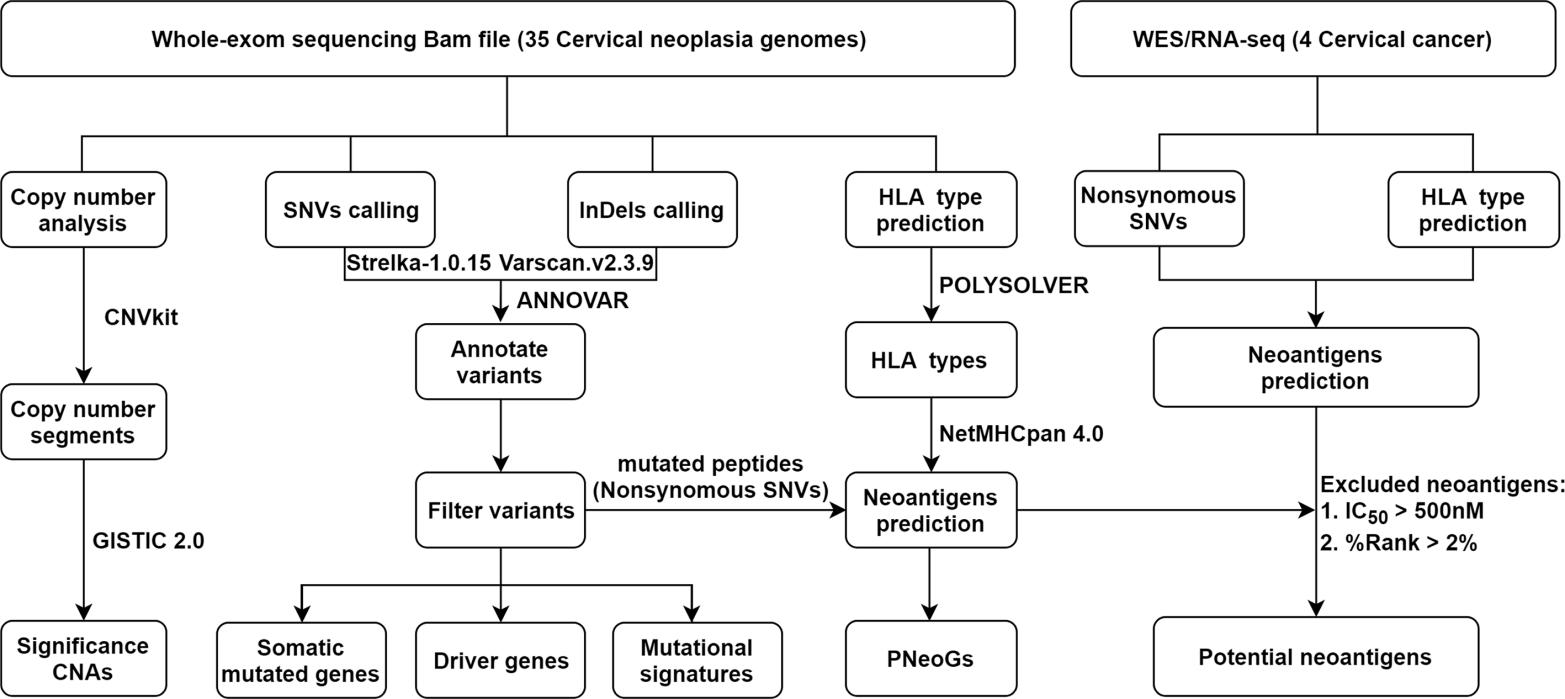
A concise flowchart of the whole-exome sequencing data analysis.

First, whole-exome sequencing was performed on 35 cervical neoplasia genomes and matched control samples, including blood cells and paired adjacent paracancer, using the Illumina HiSeq X-ten platform. In total, 99.96% of the clean reads successfully mapped to the reference genome (GRCh38 release). Each sample had at least 64 Mb of target exons covered with an average depth of 95.9**×** (range from 79.9**×** to 123**×**). More than 90.06% of the targeted regions were covered effectively with at least 30**×** reads (**Supplementary Figure 2** and **Supplementary Table 2**). A concise flowchart was schematically illustrated in **Figure 1**.

**Figure 2:**
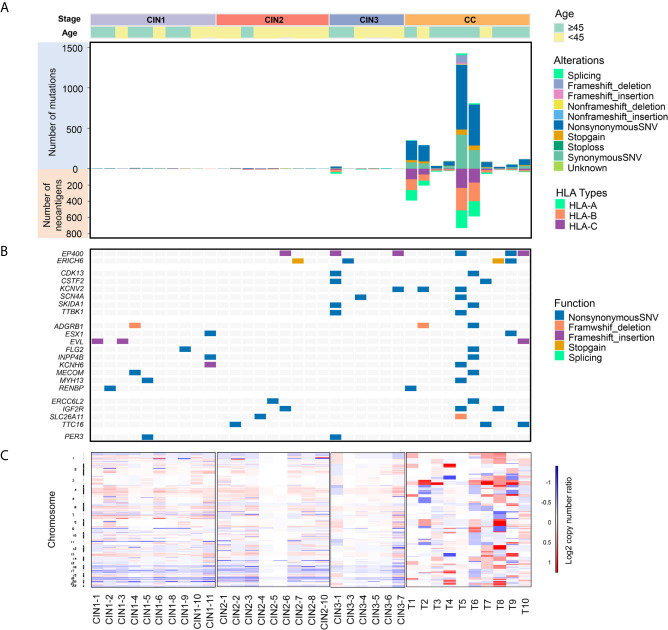
A comprehensive mutational feature of 35 cervical neoplasia genomes by whole-exome sequencing. **(A)** Upper panel: The number of all the mutations in genomes was depicted. Each bar represented an individual cervical neoplasia sample. Different colors represented different mutation types. The number of somatic mutations in CCs were significantly higher than in CINs (t-test, *p* = 6.60E-04). Lower panel: Predicted HLA class I neoantigens in 35 cervical neoplasia samples. Different colors represented different HLA types. The number of exome-derived neoantigens in CCs were significantly higher than in CINs (t-test, *p* = 3.62E-04). **(B)** A heatmap showed the genes were both detected in CINs and CCs. **(C)** DNA copy number segments of 35 cervical neoplasia patients showed that CNAs events were detected more frequently in CCs than in CINs. Red and blue boxes represented copy number amplification and deletion events, respectively (x-axis, 35 cervical neoplasia patients in each group; y-axis, chromosome number).

To identify the somatic mutations in CINs and CCs, SNVs, and Indels were called by using Strelka (24) and VarScan2 (25), respectively. In total, we detected 3,489 somatic mutations (see *Materials and Methods* section for details) in exons and splicing regions, including 2080 non-synonymous SNVs, 969 synonymous SNVs, 203 nonsense, 52 splicing, 139 frameshifts, 46 in-frameshifts. The average number of mutations was 330.5 in CCs (range from 27 to 1424), which was significantly higher than that in CIN1 (6.45), CIN2 (6.80), or CIN3 (9.67), respectively (Kruskal-Wallis chi-squared test, *P* = 8.13E-05). These results showed that the number of somatic mutations was distinctly increasing from CINs to CCs (**Figure 2A**, upper panel and **Supplementary Table 3**).

Furthermore, we analyzed the 2474 mutations involving in 2125 genes, including 2080 non-synonymous SNVs, 203 nonsense, 52 splicing and 139 frameshifts, 95.1% of which (2,363 mutations involving in 2,021 genes) were common with those reported in the TCGA-CESC data set. Among the mutations, 94.62% (2,341/2,474) of them were detected only in CCs, which indicated that the genomic alterations mainly occurred in CCs. Notably, 63 mutations in 22 genes were both observed in CINs and CCs. To validate our findings, we compared these mutated genes with those in other databases, such as COSMIC, TCGA-CESC data set, and COMSIC-CGC. The results showed that these mutated genes were all overlapped in COSMIC and TCGA-CESC, while only three mutated genes were in COMSIC-CGC database (**Figure 2B** and **Supplementary Table 4**). *EP400*(6/35) and *ERICH6*(4/35) were observed in CIN2, CIN3, and CCs, as well as *CDK13*, *CSTF2*, *KCNV2*, *SCN4A*, *SKIDA1*, and *TTBK1* were observed in CIN3 and CCs. All of these genes were detected in a consecutive group, which was a novel discovery in this cohort that was never prominently reported in previous studies (13–16). These genes in CINs and CCs, such as *EVL*, *INPP4B*, *IGF2R*, and *ADGRB1*, were involved into some well-defined cancer-related pathway, including RTK/RAS/PI(3)K, Wnt/β-catenin, p53/mTOR, cell cycle, and DNA damage, suggested that these mutated genes might be novel biomarkers for the early diagnosis and treatment of CC (**Supplementary Table 5**). Also, there were 70 mutations involving 70 genes in CIN1 or CIN2 or CIN3, while not in CCs. *IKBKB*, *TNFRSF10A*, and *IL1R1* were involved into the apoptosis-related pathways, suggesting that these mutated genes in CINs might be associated with early apoptosis events (**Supplementary Table 5**). The sequence quality, including all mutant sites, was carefully validated by integrative genomics viewer (IGV) (35) according to the criterion for variation calling (**Supplementary Figure 3** and **Supplementary Table 4**).

Meanwhile, we also analyzed DNA copy number alterations (CNAs) segments by using WES data and identified 6, 6, 3, and 14 CNAs in CIN1, CIN2, CIN3, and CCs, respectively. These results also implied that the genomic instability and alterations had a stepwise increase during the development of CC and suggested that CNAs may also occur in CINs stage. In addition, we found that significant gains occurred in chromosomes 1q, 3q, 13p, and 19q, while there are significant losses in chromosomes 6p and 16q in CCs. Our results were consistent with previous reports (13, 15–17) (**Figure 2C** and **Supplementary Figure S4**).

### Mutational Signatures in CINs and CCs

**Figure 3:**
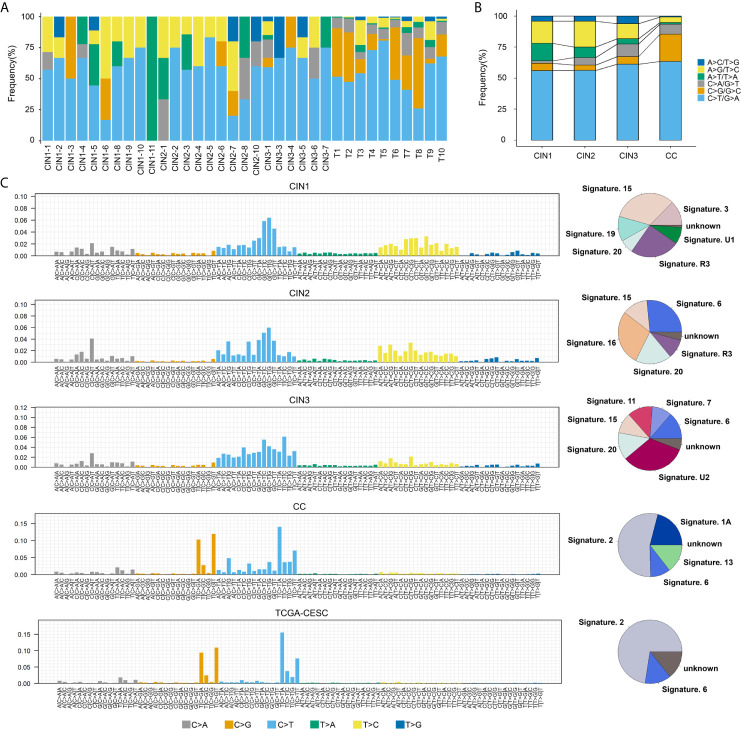
The mutation spectrums and mutational signatures in CINs and CCs. **(A)** Mutation substitution types identified the 6 substitutions classification on the basis of the nucleotide frequency of the human genome among all samples. The horizontal axes indicated the cervical neoplasia patients ID, and the vertical axes depicted the percentage of mutations categories to a specific mutation type. C>T/G>A transitions were predominant across most of cervical neoplasia patients. The horizontal axis indicated the sample ID, and the ordinate corresponds to the number of mutation types. **(B)** The trend of each nucleotide substitution subtype in CIN1, CIN2, CIN3, and CCs. **(C)** Mutational signatures in CIN1, CIN2, CIN3, CC, and TCGA-CESC, receptively. Proportion of mutation signatures distribution in each group (on the left). Inset pie chart shows the proportion of mutation signatures in each group (on the right).

In this study, we found that C>T/G>A transitions and C>G/G>C transversions dominated the mutation spectrum in both CINs (CIN1: C>T/G>A-56%, C>G/G>C-6%; CIN2: C>T/G>A-56.25%, C>G/G>C-4.17%; CIN3: C>T/G>A-61.22%, C>G/G>C-6.12%) and CCs (C>T/G>A-63.47%, C>G/G>C-21.85%) (**Figure 3A**). Interestingly, in the development process from CINs to CCs, the frequency of C>A/G>T, C>T/G>A, and C>G/G>C displayed a trend of gradual increase, while the frequency of A>T/T>A, A>C/T>G, and A>G/T>C displayed a trend of gradual decline (**Figure 3B**). C>T/G>A transition was the highest-frequency substitution type in CCs. These results indicated that the occurrence and development of cervical cancer may be closely related to C or G base damage in the genome caused by some etiologies, which are supported by previous reports (13, 15, 16).

It is well known that the diversity of mutational processes underlying the development of cancer, with potential implications for understanding of cancer aetiology, prevention, and therapy, and certain mutational signatures reveal the specific of mutational processes and the underlying aetiologies of cancer development (36). The identified mutational signatures were signature 15 (32.9%)/19 (12.7%), signature 6 (26.5%)/16 (28%), signature 6 (13.6%)/20(13.7%), and signature 2 (54.2%)/6 (10.2%) for CIN1, CIN2, CIN3, and CCs, respectively (**Figure 3C**). Signature 6 was detected in CIN2, CIN3, and CCs, while not in CIN1, suggesting that signature 6 may be of a novel biomarker for CC early warning. In contrast, the overall mutational signatures in CCs were signature 2 (54.2%) and signature 6 (10.2%), which were highly consistent with the TCGA-CESC data set [signature 2 (72.5%)/6 (13.2)] (**Figure 3C**). Signature 2 is due to over activity of members of the APOBEC family of cytidine deaminases, which convert cytidine to uracil, coupled to activity of the base excision repair and DNA replication machineries (36). We detected that signature 2 was observed notably in CCs, but not in CINs. Indeed, it has been reported that APOBEC cytidine deaminases displayed are strongly associated with cervical cancer in the previous reports (13, 15, 37). In all, these findings indicated that signature 2 was a predominant source of mutation signatures in CCs, which was dramatically distinct from CINs.

**Figure 4:**
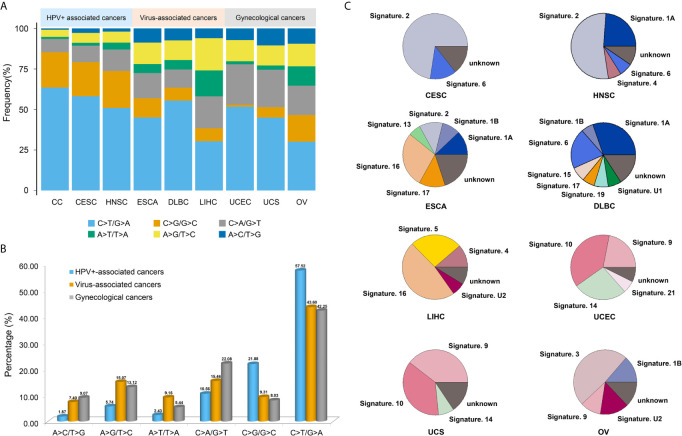
The mutation spectrums and mutational signatures in three different cancer types. **(A)** Barplots showed the proportion of each substitution subtype in HPV^+^-associated cancers, virus-associated cancers and gynecological cancers. **(B)** The average proportion of each substitution subtype in three different cancer types. **(C)** The proportion of each mutational signatures in HPV^+^-associated cancers, virus-associated cancers and gynecological cancers.

In addition, we compared the mutation spectrums and mutational signatures with two HPV^+^-associated cancers (CESC and HNSC), three virus-associated cancers (ESCA, DLBC, and LIHC), and three gynecological cancers (UCEC, UCS and OV), which were from TCGA repositories. It showed that C>T/G>A transitions (57.52%) and C>G/G>C transversions (21.88%) accounted for the highest proport in HPV^+^-associated cancers, C>T/G>A transitions (43.6%), C>A/G>T transversions (15.46%), and A>G/T>C transitions (15.07%) accounted for the highest proport in virus-associated cancers, and C>T/G>A transitions (42.25%) and C>A/G>T transversions (22.08%) accounted for the highest proportion in gynecological cancers (**Figures 4A, B**). Also, C>T/G>A transitions was the highest proportion in three different cancer types. Besides, the average proportion of C>T/G>A transitions and C>G/G>C transversions in HPV^+^-associated cancers were much higher than those in virus-associated cancers and gynecological cancers, whereas the proportion of C>A/G>T transversions in gynecological cancers was higher than that in HPV^+^-associated cancers and virus-associated cancers (**Figure 4B** and **Supplementary Table 6**). Moreover, signature 2 accounted for a relatively large proportion in HPV^+^-associated cancers, but not in virus-associated cancers and gynecological cancers, which may be a novel biomarker for the screening and treatment in HPV^+^-associated cancers. However, there were no similar signatures in virus-associated cancers or gynecological cancers (**Figure 4C**). These results suggested that different cancer types showed the different mutation spectrums and mutational signatures.

### Potential driver genes in CCs

We analyzed significantly somatic mutations on 35 cervical neoplasia genomes and identified three driver genes (**Table 1**) according to a filtering criterion (see *Materials and Methods* section for details). *PIK3CA* and *ARHGAP5* were observed in the previous reports for CC (13–16), while *ADGRB1* was a novel driver gene in CC. Notably, there were no driver genes were detected in CINs.

**Table 1:** Identifying the potential driver genes.

Sample-ID	Gene	Genome position (HG38)	CDS Mutation	Predicted by SIFT	Predicted by PolyPhen2.0	Predicted by MutationTaster	Present in CGC	Reported in previous research	Frequency in Normal	Frequency in Tumor	Reads depths
T4	*PIK3CA*	chr3:179218294	c.G1624A	0.016(D)	0.912(D)	1(D)	Yes	Yes	0.73%	35.00%	2%,1+,0-
											
T5	*PIK3CA*	chr3:179198937	c.C112T	0.072(T)	0.996(D)	1(D)	Yes	Yes	5.26%	37.86%	38%,28+,12-
T6	*PIK3CA*	chr3:179221146	c.G2176A	0.808(T)	0.396(B)	1(D)	Yes	Yes	0.00%	9.71%	10%,5+,5-
T7	*PIK3CA*	chr3:179218303	c.G1633C	0.002(D)	0.734(P)	1(D)	Yes	Yes	0.58%	11.20%	11%,11+,7-
T9	*PIK3CA*	chr3:179234297	c.A3140T	1(T)	0.07(B)	1(D)	Yes	Yes	0.00%	30.39%	27%,14+,2-
T10	*PIK3CA*	chr3:179199088	c.G263A	0.054(T)	0.971(D)	1(D)	Yes	Yes	9.63%	80.42%	80%,40+,75-
T2	***ADGRB1***	chr8:142542406	c.4172_4175del	.	.	.	NO	NO	0.00%	10.39%	–
T6	***ADGRB1***	chr8:142542106	c.C3872T	0.005(D)	0.966(D)	0.976(D)	NO	NO	0.00%	17.50%	18%,9+,5-
T5	*ARHGAP5*	chr14:32092620	c.G1951A	0.603(T)	0.008(B)	1(D)	Yes	Yes	3.31%	41.32%	41%,8+,12-
T6	*ARHGAP5*	chr14:32091046	c.C377G	.	.	1(A)	Yes	Yes	0.00%	11.43%	11%,2+,6-
T10	*ARHGAP5*	chr14:32091085	c.G416A	0.001(D)	0.997(D)	1(D)	Yes	Yes	2.00%	44.23%	45%,25+,27-

D, deleterious (sift< = 0.05); T, tolerated (sift> 0.05).

D, probably damaging (> = 0.909); P, possibly damaging (0.447<=pp2_hvar< = 0.909); B, benign (pp2_hvar< = 0.446).

A, disease_causing_automatic; D, disease_causing; N, polymorphism; P, polymorphism_automatic.

*PIK3CA* (phosphatidylinositol 3-kinase catalytic subunit alpha), one of the most frequently mutated genes, was only detected in CCs. In our current study, *PIK3CA* point mutations were detected in 60% (6/10) CCs, which was higher than that reported in previously genome sequencing studies [27.3% in TCGA-CESC data set; 26% in Cancer Genome Atlas Research Network et al. (15); 12.6% in Ojesina et al. (13); 16.67% in Huang et al. (16); 53.33% in Chung et al. (17)]. Moreover, mutations in *PIK3CA* were detected at commonly observed nucleotide positions R88Q, E545Q and H1047L in three CCs (38). Expect the substitutions R38C, all of the mutations in *PIK3CA* had been reported in cervical adenocarcinoma in the COSMIC database_v90 (https://cancer.sanger.ac.uk/cosmic/). *ARHGAP5* (Rho GTPase activating protein 5) was identified only in CCs with 30% (3/10) mutation ratio, of which all mutation sites had already been reported in COSMIC, as well as, two mutations (G1951A and G416A) were probably damaging (**Table 1**). *ADGRB1* (adhesion G protein-coupled receptor B1), a newly recognized driver gene in CCs with 20% (2/10) mutation ratio, of which one mutation was probably damaging (**Table 1**). COSMIC database showed that *ADGRB1* was a moderately over-expressed in 6.84% (21/307) of CCs, and there were many types of variants, including somatic mutations, CNVs and dysregulation in *ADGRB1* in a quantity of cancers. Similar to the previous reporting the driver genes in CC (13, 15, 16), *FBXW7*, *EP300*, *CASP8* and *FAT1*(10%, 20%, 10% and 10% of tumor, respectively) were also identified in our cohort with a low frequency, which may result from a small sample size in our cohort.

### Discovery the Neoantigens in Both CINs and CCs

To obtain more data to identify neoantigens, we downloaded both WES data and RNA-seq data from four pairs of CC samples (16). Of which the four WES data was also used to predict the neoantigens, while the RNA-seq data were used to examine the expression of neoantigens.

Then we performed POLYSOLVER to identify the four-digit HLA class I alleles in 39 samples (4 samples plus 35 samples). We found that HLA-A*26:01and HLA-C*07:02 were the most common alleles at HLA-A and HLA-C loci in both CCs and CINs (CCs-HLA-A: 42.86%, CCs-HLA-C: 42.86%; CINs-HLA-A: 20%, CINs-HLA-C: 20%). For HLA-B, the most prevalent alleles were HLA-B*15:18 (35.71%) in CCs, while HLA-B*40:01 (12%) was the most prevalent allele in CINs (**Table 2**).

**Table 2:** Number of neoantigens, nonsynonymous mutations and HLA class I allotypes of 39 patients.

Sample-ID	Number of neoantigens	Number of nonsynomous mutations	HLA-A*	HLA-B*	HLA-C*	Sequencing strategies
CIN1-1	0	3	/			WES
CIN1-2	1	3	–	40:01	12:03	WES
CIN1-3	1	1	–	–	07:02	WES
CIN1-4	3	3	26:01	–	07:02	WES
CIN1-5	0	7	/			WES
CIN1-6	1	3	11:01	–	–	WES
CIN1-8	2	2		07:02/67:01		WES
CIN1-9	2	1	26:01	–	–	WES
CIN1-10	0	1	/			WES
CIN1-11	2	1	26:01	67:01	–	WES
CIN2-1	0	2	/			WES
CIN2-2	3	3	–	37:01	–	WES
CIN2-3	12	6	03:01	07:03	14:02	WES
CIN2-4	10	3	26:01	40:01	07:02	WES
CIN2-5	7	5	03:01/03:02	–	07:02	WES
CIN2-6	2	1	03:02	–	–	WES
CIN2-7	1	3	24:02	–	–	WES
CIN2-8	2	2	–	40:01	–	WES
CIN2-10	0	3	/			WES
CIN3-1	61	16	02:01/11:01	07:03	12:03	WES
CIN3-3	0	2	/			WES
CIN3-4	10	1	23:01	15:01	07:02/12:03	WES
CIN3-5	6	2	26:01	15:18/37:01	12:02	WES
CIN3-6	3	2	–	55:02	–	WES
CIN3-7	0	0	/			WES
T1	392	241	26:01	15:18/67:*	12:*	WES
T2	209	199	26:01	15:18	12:03	WES
T3	36	26	26:01	15:18/55:02	12:03	WES
T4	29	54	26:01	40:01	07:02	WES
T5	732	800	34:01	15:18/67:01	07:02	WES
T6	589	504	03:01	40:01	07:02	WES
T7	64	33	11:01	37:01	12:03	WES
T8	12	15	01:02	40:01	07:02	WES
T9	20	61	26:01	15:18	07:02	WES
T10	42	71	26:01	07:02	07:02	WES
CC-H024	155	77	02:07/02:01	35:01	03:03	WES/RNA-seq
CC-H027	5	40	02:07	52:01	01:02	WES/RNA-seq
CC-X004	134	129	02:01	52:01	01:02	WES/RNA-seq
CC-X008	38	28	31:02	40:06	03:04/14:02	WES/RNA-seq

*List separator.

**Figure 5:**
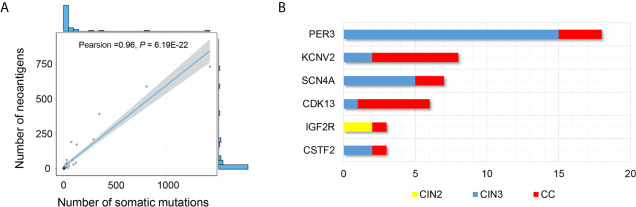
Distribution of potential neoepitopes genes during stepwise cervical carcinogenesis. **(A)** Correlation between the number of mutations and the number of exome-derived neoepitopes (Pearson’s correlation coefficient, *r* = 0.96, *P* = 6.19E-22). **(B)** Barplots showed the number of PNeoGs in both CINs and CCs.

Moreover, we performed NetMHCpan 4.0 program to predict the neoantigens using the parameters of %Rank < 2% and IC50 <500 nM. We identified 2,586 potential neoantigens in CINs and CCs (**Supplementary Table 7**). CINs had a median of 3.1 non-synonymous mutations (range, from 1 to 16) with a mean of 5.3 neoantigens (range, from 1 to 61), whereas CCs had a higher mutation burden (median, 162.7; range, from 15 to 800) with a mean of 175.5 neoantigens (range, 5 to 732) (**Table 2** and **Figure 2A**, lower panel). The number of non-synonymous mutations showed a strong linear correlation with the number of neoantigens (Pearson’s correlation coefficient *r* = 0.96, *P* = 6.19E-22) (**Figure 5A**) that were consistent with previous research on TCGA-CESC data set (Pearson’s correlation coefficient *r* = 0.9, *P <*0.001) (39).

In addition, we identified 6 “potential neoantigens genes” (PNeoGs) in both CINs and CCs: five PNeoGs in both CIN3 and CCs, one PNeoG in both CIN2 and CCs (**Figure 5B** and **Supplementary Table 7**). For example, *IGF2R* was an immune-related gene (IRGs), deposit in the Immunology Database and Analysis Portal (ImmPort). Besides, it was reported that *IGF2R* has an oncogenic role through transportation mannose-6-phosphate (M6P)-tagged in CCs (40). These results indicated that neoantigens could be generated in the CIN stage. If it is the case, neoantigen-based immunotherapeutic measures might be useful for prevent of CC.

It is one interesting question whether the genes corresponding to these neoantigens are expressed? To answer this question, we used the RNA-seq data to examine the expression of neoantigens. The result showed that there were 332 potential neoantigens in four pairs of samples, 50.9% (167/332, 167 neoantigens involving in 59 genes) of which were expressed (**Supplementary Table 8**).

### Validation the Potential Neoantigens by Public Data

To verify the accuracy of our results and to discover the potential immune therapeutic targets, we compared our results with Tumor-Specific Neoantigen database (TSNAdb) (41), Immune Epitope Database (IEDB) (42), and CTDatabase (43). Out of 2586 potential neoantigens, 45 neoantigens were overlapped in the three databases. In which 10 neoantigens involving into five proteins was in TCGA-CESC data set (TSNAdb), two neoantigens involving into two proteins in IEDB, and 33 neoantigens involving into 11 proteins in CTAs. Out of 18 neoantigen-related proteins, six proteins were reported to the therapeutic targets and the corresponding drugs in Therapeutic Target Database (44). For example, *PIK3CA* was an inclusion criterion in one clinical trial (NCT02957266) for directing cervical carcinoma therapy (45), as well as, *ERBB3* and *FBXW7* were considered as biomarkers in several clinical trials for malignant solid tumor (**Table 3**).

**Table 3:** A list of candidate neoantigens validated by three public databases.

No	Sample	Identity	Protein	Mutation AA	HLA types	Affinity(nM)	%Rank	Validated	Drugs
1	T9	LHGGWTTKM	PIK3CA	H1047L	HLA-B15:18	490.2144	0.1622	TCGA-CESC	BAY 80-6946
2	T5	LRMVRGTQVY	ERBB3	V104M	HLA-C07:02	301.045	0.0906	TCGA-CESC	Trastuzumab
3	T5	LRMVRGTQV	ERBB3	V104M	HLA-C07:02	192.3883	0.1237	TCGA-CESC	Elisidepsin
4	T5	RMVRGTQVY	ERBB3	V104M	HLA-B15:18	265.2144	0.0758	TCGA-CESC	EZN-3920
5	T1	MIDSKTAEM	DSG3	I444M	HLA-B67:*	158.4915	0.6763	TCGA-CESC	Literature-reported Target
6	T6	ALPASERGWK	C3orf70	S6L	HLA-B40:01	370.518	0.9879	TCGA-CESC	/
7	T5	SYTHIQYLF	SH2D3C	Q129R	HLA-A34:01	283.251	0.1863	IEDB	/
8	T2	LTFRDVAIEF	ZNF468	Q19E	HLA-C12:03	358.2076	1.2376	IEDB	/
9	CC-X004	LHVLMGHVAAV	FBXW7	R505G	HLA-A02:01	25.157	0.3445	TCGA-CESC	Literature-reported Target
10	CC-X004	HVLMGHVAAV	FBXW7	R505G	HLA-A02:01	26.1568	0.3571	TCGA-CESC	
11	CC-X004	VLMGHVAAV	FBXW7	R505G	HLA-A02:01	82.6567	0.9305	TCGA-CESC	
12	CC-X004	VLMGHVAAV	FBXW7	R505G	HLA-A02:01	3.1125	0.0094	TCGA-CESC	
13	T5	HTKDIFNVK	ADAM29	R399H	HLA-A34:01	63.2156	0.0205	CTAs	/
14	T5	MASFRKLML	LUZP4	T8M	HLA-B67:01	418.5688	0.615	CTAs	/
15	T5	FPNLPHLSF	MAGEC3	R9H	HLA-C07:02	468.0406	0.1553	CTAs	/
16	T5	FPNLPHLSF	MAGEC3	R9H	HLA-B67:01	11.2897	0.0146	CTAs	/
17	T5	MPLFPNLPHL	MAGEC3	R9H	HLA-B15:18	10.6791	0.013	CTAs	/
18	T5	SPIEIGLFI	OTOA	T195M	HLA-A34:01	52.8519	0.1197	CTAs	/
19	T5	SRHNKALKL	OTOA	T195M	HLA-C07:02	75.4928	0.0382	CTAs	/
20	T5	FPKLTKNML	PRSS55	A236V	HLA-B15:18	32.3202	0.0684	CTAs	/
21	T5	MFPKLTKNM	PRSS55	A236V	HLA-B15:18	374.1032	0.2469	CTAs	/
22	T5	MFPKLTKNML	PRSS55	A236V	HLA-A34:01	442.851	0.6403	CTAs	/
23	T1	IPALSARDL	SAGE1	M193L	HLA-A26:01	78.8728	0.1714	CTAs	/
24	T1	LINMAATPI	SAGE1	M193L	HLA-B15:18	228.4982	0.9079	CTAs	/
25	T1	MAATPIPAL	SAGE1	M193L	HLA-B15:18	37.04	0.0829	CTAs	/
26	T1	MAATPIPAL	SAGE1	M193L	HLA-B15:18	12.4152	0.0368	CTAs	/
27	T1	SARDLYATV	SAGE1	M193L	HLA-B15:18	32.9825	0.1358	CTAs	/
28	T1	TPIPALSARDL	SAGE1	M193L	HLA-A26:01	103.2219	0.2134	CTAs	/
29	T6	IVKNDLIAK	SPAG9	E283K	HLA-B40:01	147.4087	0.5079	CTAs	/
30	T6	KVDKLTCEK	SPAG9	E283K	HLA-A03:01	261.5526	0.7731	CTAs	/
31	CC-X004	SVMKLCLIMV	AKAP4	A712V	HLA-A02:01	91.7051	1.0019	CTAs	/
32	CC-H024	TPAMEGAVA	ARX	V508M	HLA-B35:01	440.195	0.8127	CTAs	Cetuximab PEGylated IFN beta 1-a HGH-CTP
33	CC-H024	LLRQPTPAM	ARX	V508M	HLA-C03:03	135.4831	0.4402	CTAs	
34	CC-H024	MALLLVLFLV	CRISP2	P5L	HLA-A02:01	223.9094	1.7662	CTAs	/
35	CC-H024	LLLVLFLVTV	CRISP2	P5L	HLA-A02:01	184.453	1.5646	CTAs	/
36	CC-H024	ALLLVLFLV	CRISP2	P5L	HLA-A02:01	47.3197	0.6001	CTAs	/
37	CC-H024	LLVLFLVTV	CRISP2	P5L	HLA-A02:01	131.0198	1.2594	CTAs	/
38	CC-H024	VLFLVTVLL	CRISP2	P5L	HLA-A02:01	106.6147	1.0997	CTAs	/
39	CC-H024	FLVTVLLPS	CRISP2	P5L	HLA-A02:01	32.8486	0.4379	CTAs	/
40	CC-H024	MALLLVLFL	CRISP2	P5L	HLA-C03:03	243.1628	0.6375	CTAs	/
41	CC-H024	LVLFLVTVL	CRISP2	P5L	HLA-C03:03	461.3886	0.9529	CTAs	/
42	CC-H024	KVWVQGHYL	MAGEC2	R287Q	HLA-A02:01	224.0936	1.7671	CTAs	CV-9201
43	CC-H024	WVQGHYLEY	MAGEC2	R287Q	HLA-B35:01	32.875	0.1302	CTAs	/
44	CC-H024	EVPHSSPPY	MAGEC2	R287Q	HLA-B35:01	261.5695	0.591	CTAs	/
45	CC-H024	VPHSSPPYY	MAGEC2	R287Q	HLA-B35:01	20.2	0.0829	CTAs	/

*List separator.

Interestingly, we found that almost all of immune targets occurred only in one sample. To validate the result, we analyzed the frequency of potential neoantigens in the similar data sets, such as TCGA-CESC. The result showed that there were 7,748 neoantigens in a total of 286 samples, only 49 neoantigens appeared simultaneously in at least two samples, and nine neoantigens appeared simultaneously in three samples. These results were basically consistent with our results. In addition, only 10 of the identified neoantigens in our study overlapped with those in the TCGA-CESC database (**Supplementary Table 9**). It suggested that neoantigens have a large heterogeneity.

Taken together, respective public data validation results indicated a list of immune therapeutic targets, which can narrow the scope of immunological targets for cervical cancer immunotherapy. Due to individual differences, almost all patients in our cohort have found no neoantigens in common, which indicates that personalized immunotherapy maybe one of the most effective immunotherapies for cervical cancer.

## Discussion

In this study, we performed a comprehensive analysis for the genomic variation profiling to identify potential neoantigens, which were taken as potential immune therapeutic targets for cervical cancer. This study is the first time to analyze the neoantigens in CINs and CCs by whole-exome sequencing.

Somatic mutations analysis revealed that genomic alterations occurred during the development from CINs to CCs, and 22 high frequently mutated genes were identified in both CINs and CCs. *EP400*, a known component of multiple histone acetylase complexes, was regarded as an E2-dependent regulator of human papillomavirus oncogene expression (46). *PER3* was reported to be related to cancer development (47, 48). Meanwhile, we also observed that the CNA segments in the CCs were more susceptible than in CINs. Intriguingly, the mutation spectrum in CINs was the same as that in CCs, whereas mutation signatures revealed different mutational processes dominant in the progression of CC stages. Consistent with previous report (15), we identified signature 2, an APOBEC-related signature, was only observed in CCs. Notably, signature 6, in association with defective DNA mismatch repair, was detected in CIN2, CIN3, and CCs, but not in CIN1, indicating that signature 6 may be a novel biomarker for the early diagnosis and treatment of CC.

Three potential driver genes were identified in this study, including *PIK3CA*, *ARHGAP5*, and *ADGRB1*. Especially, *ADGRB1* as a novel driver gene. *PIK3CA* was one of the most frequently mutated genes associated with cervical carcinoma and had been observed in our cohort. *PIK3CA* was only observed in CCs and was much higher than that reported in previously genome sequencing studies [14% in Ojesina et al. (13), 16.67% in Cancer Genome Atlas Research Network (15), 26% in Huang et al. (16)]. *ADGRB1* identified in CCs was a newly recognized driver gene that was found in Aflatoxin-Associated Hepatocellular Carcinoma (49), suggesting that *ADGRB1* play a vital role in cancer pathogenesis.

Tumor immunotherapy is a treatment method to control and eliminate tumors by restarting and maintaining the tumor-immune microenvironment and restoring the normal anti-tumor immune response. Neoantigens are important targets of immunotherapy, including monoclonal antibody immune checkpoint inhibitors, therapeutic antibodies, cancer vaccines, cell therapy, and small molecule inhibitors. In recent years, tumor immunotherapy has been gaining good news. Currently, it has demonstrated strong anti-tumor activity in the treatment of solid tumors, such as melanoma, non-small cell lung cancer, kidney cancer, and prostate cancer, and several tumor immunotherapies drugs have been approved for clinical application by the US Food and Drug Administration (FDA) (4, 50). However, the use of genetic mutation information to identify neoantigens of CINs and CCs is limited. In this study, we identified 2586 neoantigens during the stepwise process from CINs to CCs and found that the number of exome-derived neoantigens in CCs were significantly higher than that in CINs. These data also indicated that some of the neoantigens were occurred in the early stage of CCs, suggesting that the progression of cervical neoplasia genomes from CINs to CCs may affect the tumor microenvironment and lead to cancer development. One of the PNenGs, *IGF2R*, was a tumor suppressor gene, with a high frequency of loss-heterozygosity (LOH) and protein expression in diverse types of malignant cancer (51, 52). In particular, a recent study identified *IGF2R* as a poor prognostic biomarker for CC and demonstrated that it had oncogenic functions (40). We also identified that the somatic mutations at *IGF2R* were deemed to highly damaging and the exome-derived neoantigens were detected in both CINs and CCs. Furthermore, we also demonstrated *IGF2R* was correlated with a poor prognosis and was upregulated in tumor tissues compared with normal tissues in the TCGA-CESC data set (**Supplementary Figure 5**) (53). These results suggest that *IGF2R* expression or *IGF2R* somatic mutations and exome-derived neoantigens may be a novel biomarker for CC early warning, screening and therapy, although further extended clinical studies might be required.

Based on the present neoantigen-related public database, we identified 45 different neoantigens involving into 18 genes (**Table 3**), which were regarded to be candidate targets for CC immunotherapy. Based on the available public TCGA-CESC data set, we found that *ERBB3* and *DSG3* were significantly up-regulated at RNA levels in tumors compared to normal tissues, whereas *C3orf70*, *SH2D3C*, and *SPAG9* were down-regulated in tumors. The down-regulated *C3orf70* expression is closely related with a poor prognosis of cervical cancer (**Supplementary Figure 5**) (53). Our findings suggested that these genes could be used for neoantigen-targeted immunotherapies in CC in the future. Theoretically, the predicted neoantigens need to be synthesized and tested for reactivity by autologous T cells using various assays, such as ELISPOT, fluorescently labeled HLA tetramers, or barcode-labeled HLA multimers (54). Furthermore, we also analyzed the neoantigens derived from Indels mutations (**Supplementary Table 3**) (55). The results showed that 678 potential neoantigens involving into 142 indels were identified (**Supplementary Table 10**). Moreover, we compared these neoantigens with TSNAdb, IEDB, and CTAs database. We found that only SPAG17 protein were overlapped with CTAs, and none of them were overlapped in TSNAdb or IEDB database or the corresponding drugs in Therapeutic Target Database. In this study, because of the limited available clinical samples, we could not do the experimental verification to obtain the effective neoantigens. We will further study the mechanism of action of neoantigen-specific T cell–activating immunotherapeutic approaches in preclinical models of cervical cancer and extend the neoantigen-target immunotherapy approaches in large sample size of cervical cancer.

Up to now, the landscape of alterations and neoantigens from CINs to CCs is still unclear. One of the important reasons is clinical samples of CINs are usually small and intermixed with normal cells. By comparison with the previous study (18), our study has expanded the number of samples, especially in CIN1, to disclose genomic alterations from CIN1, CIN2, CIN3 to CCs. Furthermore, our study describes the neoantigen landscape during the stepwise processing from CINs to CCs and narrows the immune targets to provide insight for immunotherapy guidelines. Our present findings disclose an important clinical significance and cast light on the potential for early warning, diagnoses and immunotherapy in CC. Nonetheless, it is urgent to investigate a comprehensive molecular and integrative analyses for identifying attractive targets of CC immunotherapy *via* large-scale studies of genomic, transcriptomic, epigenomic, and proteomic data.

## Conclusion

In conclusion, we systematically analyzed the comprehensive genomic variation profiling for identifying the potential exome-derived neoantigens in CINs and CCs. Our findings may yield insights into the similarities and differences in genomics between CINs and CCs and provide attractive targets to increase the effectiveness of immunotherapies in CC.

## Data Availability Statement

The data sets presented in this study can be found in online repositories. The names of the repository/repositories and accession number(s) can be found below: https://www.ncbi.nlm.nih.gov/, PRJNA419579.

## Ethics Statement

The studies involving human participants were reviewed and approved by SPH-2016010. The patients/participants provided their written informed consent to participate in this study.

## Author Contributions

JH conceived the project and designed the experiments. CB, AN, and WH performed sequencing. CB and JH analyzed the sequencing data. WH performed the pathology experiments. JH contributed reagents, materials, and analysis tools. WH, HX, LX, JL, and BZ contributed the samples. CB and JH integrated, analyzed, and interpreted all data. JH contributed to the supervision of the work. CB and JH wrote the manuscript with the assistance and final approval of all authors. All authors contributed to the article and approved the submitted version.

## Funding

This study was supported by grants from the Science and Technology Development Project of Pukou District, Nanjing (S2017-10); Health Fit Technology Project of Health Commission of Xinjiang Autonomous Region (FPSYTG-201901) and Scientific Research Project of Shanghai Health Commission (201840340).

## Conflict of Interest

The authors declare that the research was conducted in the absence of any commercial or financial relationships that could be construed as a potential conflict of interest.

## Supplementary Material

Supplementary Figure 1 The result of H&E saining in 10 CC patients.

Supplementary Figure 2 Detail information about sample data quality control (QC) of 35 cervical neoplasia genomes.

Supplementary Figure 3 A heatmap showed the distribution of gene in CINs group. More detail information can be obtained in **Supplementary Table S4**.

Supplementary Figure 4 Copy number profiles, amplification and deletion. GISTIC2.0 analyses of CNAs in 35 samples revealed significantly amplification and deletion regions peaks on several chromosomes. Red and blue lines represented copy number amplification and deletion events, respectively.

Supplementary Figure 5 The expression of the neoantigen-related genes and the and prognosis informatics from GEPIA database. **(A–F)** The expression of the neoantigen-related genes. Red indicated tumor and grey indicated normal. **(G, H)** Kaplan–Meier analysis comparing the groups with a different expression of *IGF2R* and *C3orf70* in TCGA-CESC.
