# Supplementary figures and images for: Identifying Potential Neoantigens for Cervical Cancer Immunotherapy Using Comprehensive Genomic Variation Profiling of Cervical Intraepithelial Neoplasia and Cervical Cancer
Source: Front Oncol. 2021 Jun 17;11:672386. doi: 10.3389/fonc.2021.672386 (PMC8249860; doi:10.3389/fonc.2021.672386)

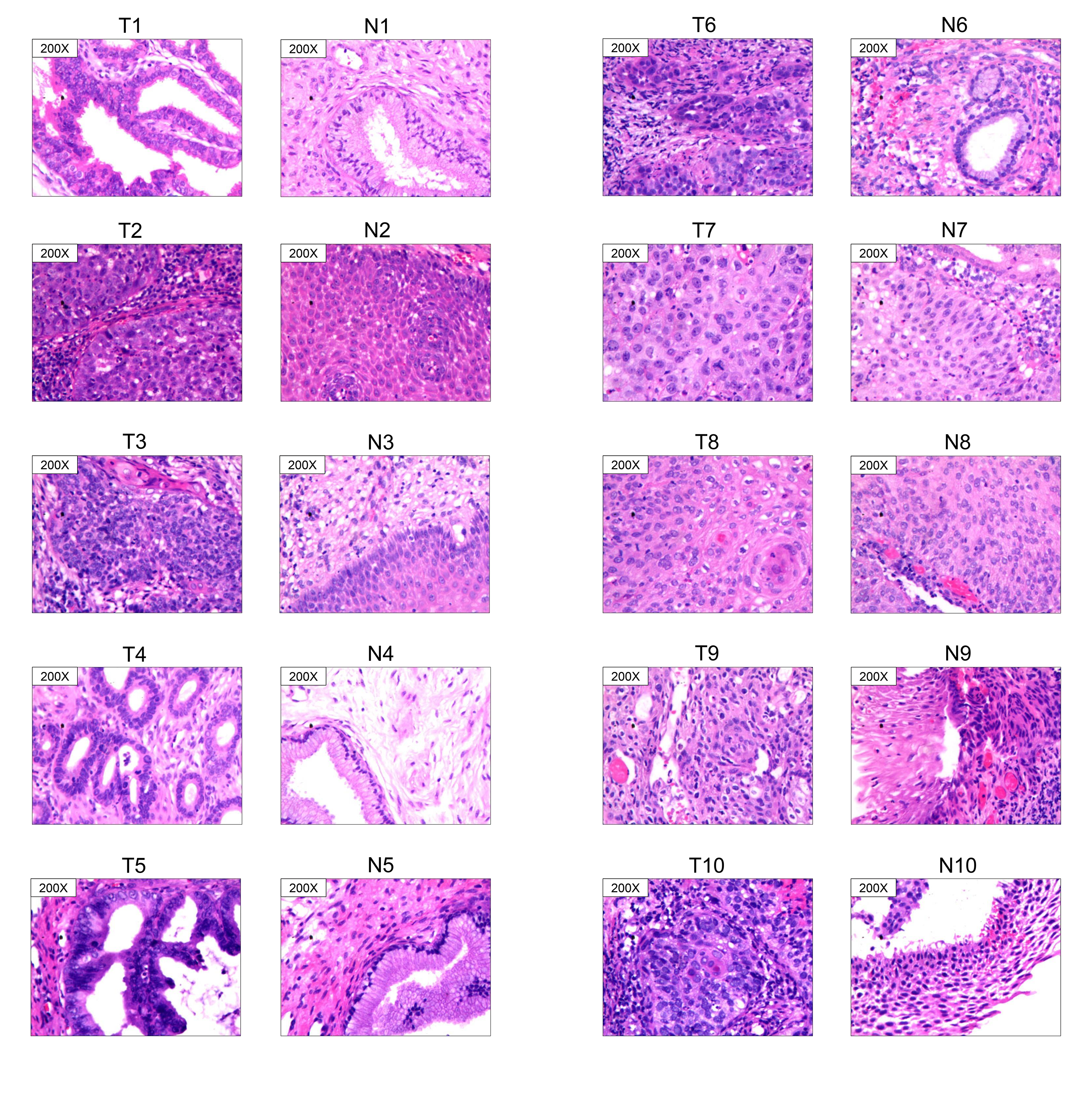

Supplement: Supplementary Figure 1 — The result of H&E saining in 10 CC patients. [file Image_1.tif]

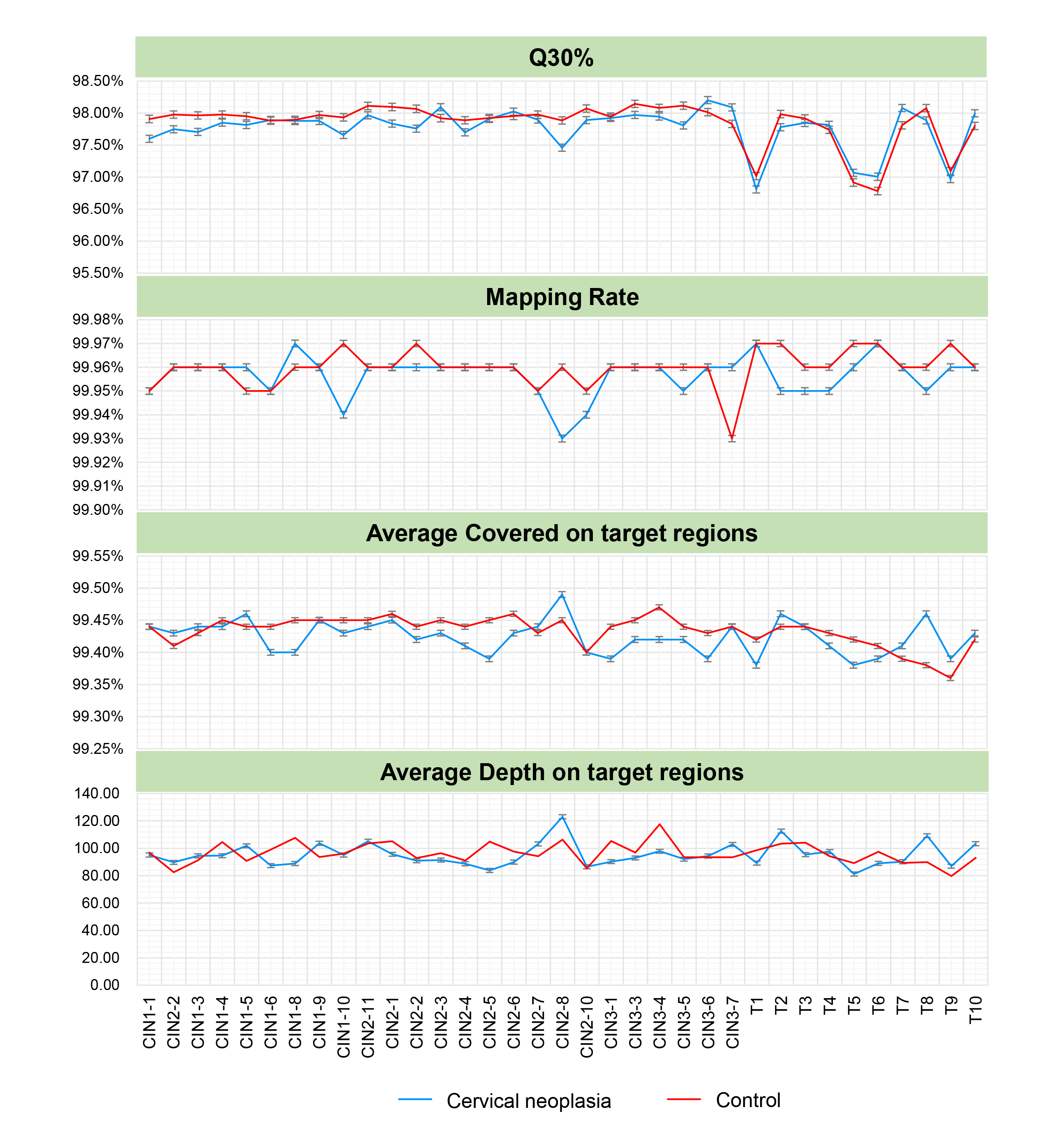

Supplement: Supplementary Figure 2 — Detail information about sample data quality control (QC) of 35 cervical neoplasia genomes. [file Image_2.tif]

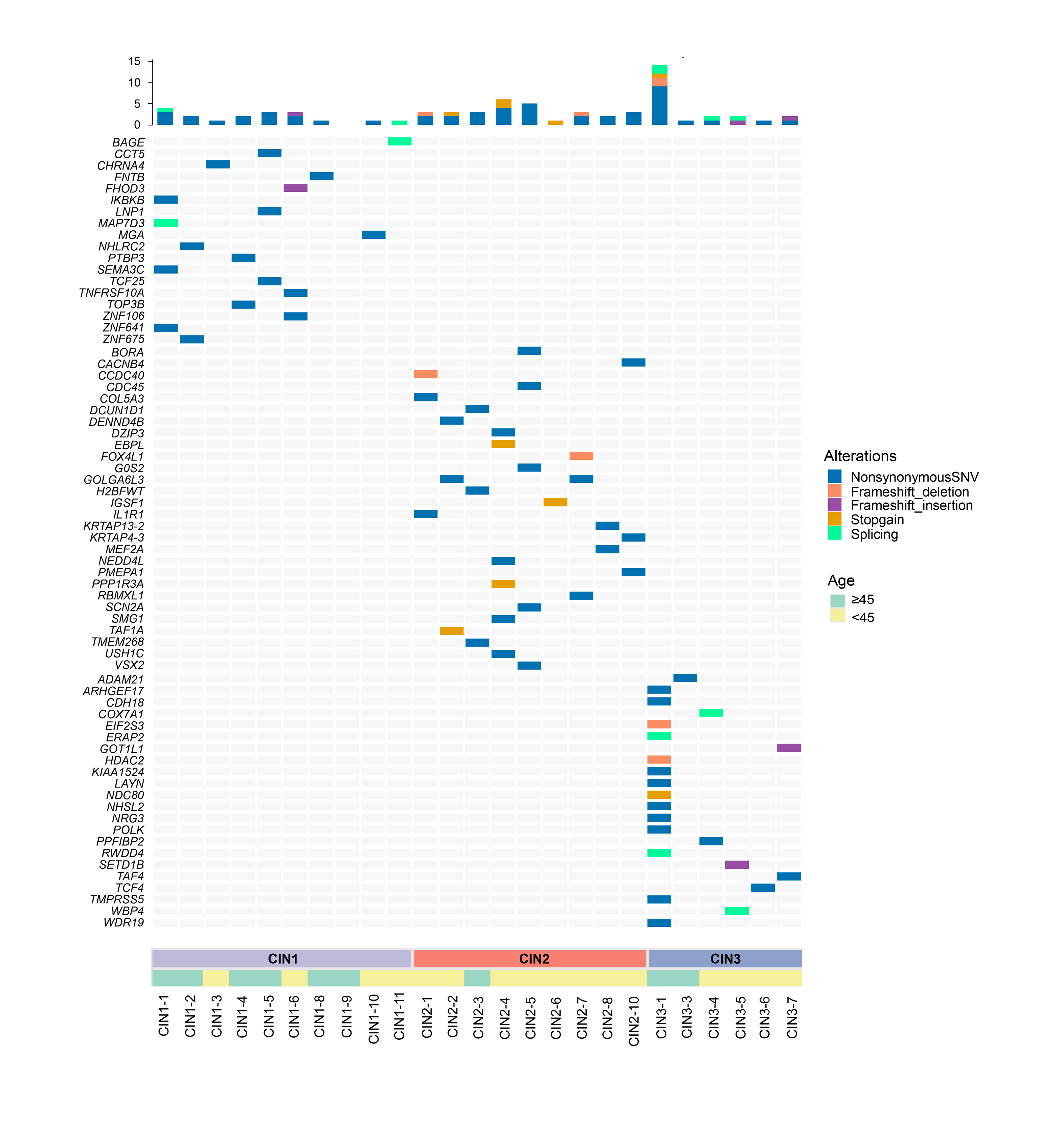

Supplement: Supplementary Figure 3 — A heatmap showed the distribution of gene in CINs group. More detail information can be obtained in Supplementary Table S4 . [file Image_3.tif]

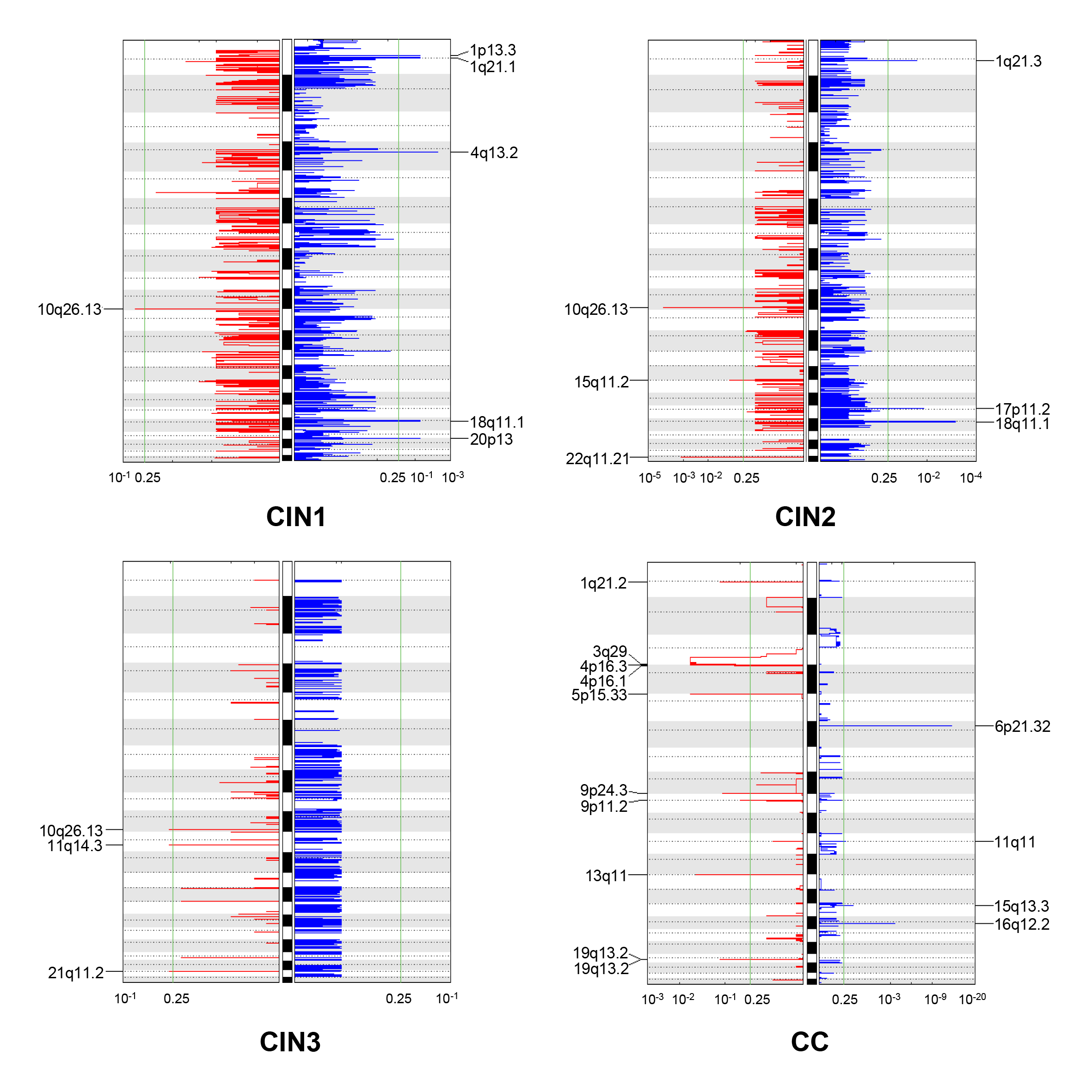

Supplement: Supplementary Figure 4 — Copy number profiles, amplification and deletion. GISTIC2.0 analyses of CNAs in 35 samples revealed significantly amplification and deletion regions peaks on several chromosomes. Red and blue lines represented copy number amplification and deletion events, respectively. [file Image_4.tif]

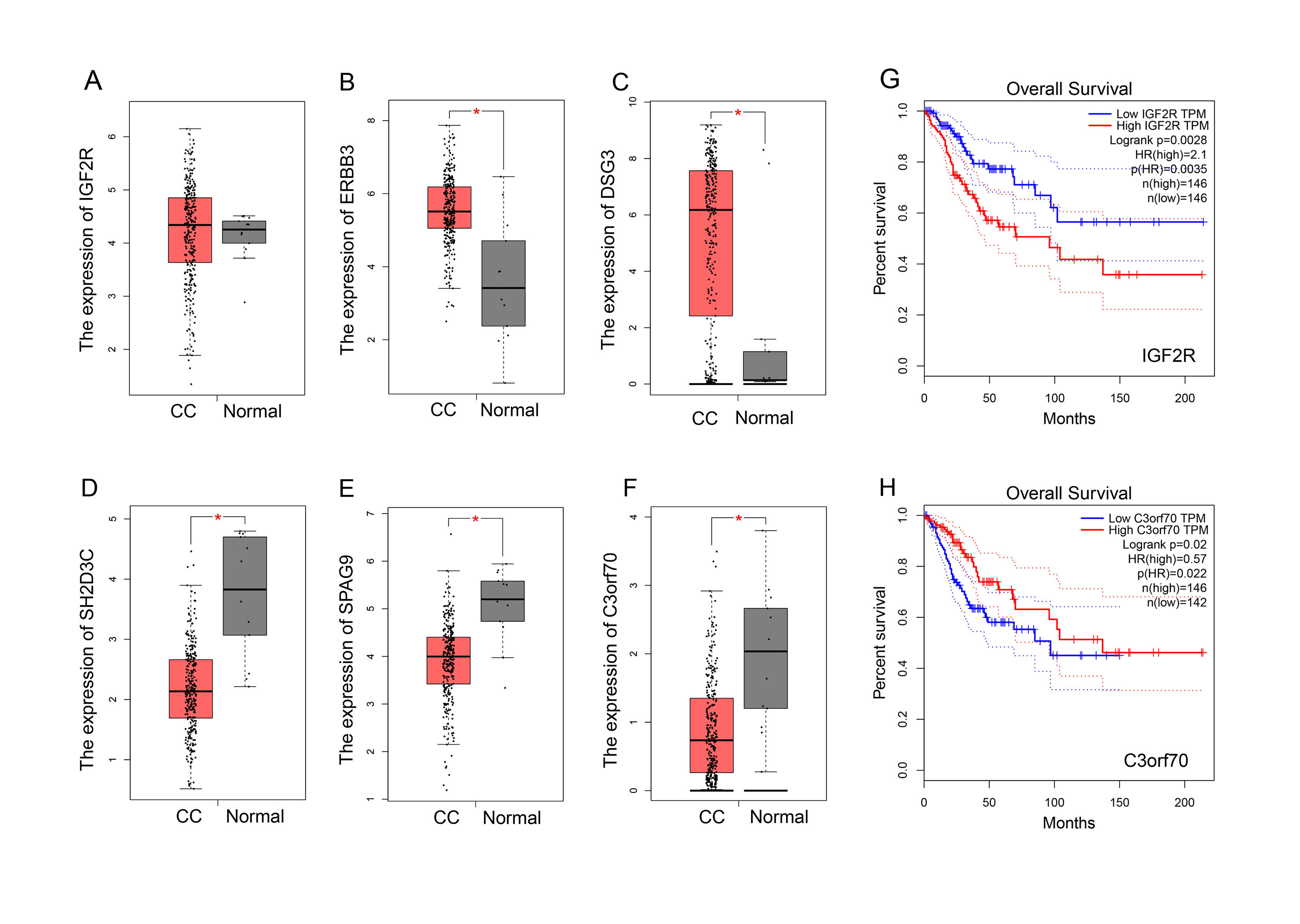

Supplement: Supplementary Figure 5 — The expression of the neoantigen-related genes and the and prognosis informatics from GEPIA database. (A–F) The expression of the neoantigen-related genes. Red indicated tumor and grey indicated normal. (G, H) Kaplan–Meier analysis comparing the groups with a different expression of IGF2R and C3orf70 in TCGA-CESC. [file Image_5.tif]
