# Supplementary material for: Identifying Potential Neoantigens for Cervical Cancer Immunotherapy Using Comprehensive Genomic Variation Profiling of Cervical Intraepithelial Neoplasia and Cervical Cancer
Source: Front Oncol. 2021 Jun 17;11:672386. doi: 10.3389/fonc.2021.672386 (PMC8249860; doi:10.3389/fonc.2021.672386)
Supplement: Supplementary file 11 [file Table_6.docx]

**Table S6 ｜ The average ratio of each substitution subtype in three different cancer types.**

| Groups | | A>C/T>G | A>G/T>C | A>T/T>A | C>A/G>T | C>G/G>C | C>T/G>A |
| --- | --- | --- | --- | --- | --- | --- | --- |
| HPV^+^-associated  cancers | CC(In this study) | 0.86% | 4.39% | 1.21% | 8.23% | 21.84% | 63.48% |
|  | TCGA-CESC | 2.79% | 6.06% | 1.79% | 10.20% | 21.00% | 58.16% |
|  | TCGA-HNSC | 1.97% | 6.76% | 4.29% | 13.25% | 22.81% | 50.92% |
| Average | | 1.87% | 5.74% | 2.43% | 10.56% | 21.88% | 57.52% |
| Virus-associated  cancers | TCGA-ESCA | 8.75% | 13.21% | 5.62% | 15.52% | 11.87% | 45.03% |
|  | TCGA-DLBC | 7.38% | 12.13% | 5.88% | 11.18% | 8.00% | 55.43% |
|  | TCGA-LIHC | 6.06% | 19.88% | 15.96% | 19.68% | 8.06% | 30.35% |
| Average | | 7.40% | 15.07% | 9.16% | 15.46% | 9.31% | 43.60% |
| Gynecological  cancers | TCGA-UCEC | 7.21% | 13.09% | 1.80% | 24.98% | 1.21% | 51.71% |
|  | TCGA-UCS | 10.54% | 12.32% | 2.61% | 23.14% | 6.48% | 44.92% |
|  | TCGA-OV | 9.48% | 13.95% | 11.92% | 18.12% | 16.41% | 30.13% |
| Average | | 9.07% | 13.12% | 5.44% | 22.08% | 8.03% | 42.25% |
